# An Adolescent Presenting With Mania and Catatonia Associated With Coronavirus Disease-2019 Encephalitis

**DOI:** 10.7759/cureus.51829

**Published:** 2024-01-07

**Authors:** Sahar Ashrafzadeh, Narges Hosseini, Fatemeh Moharreri, Shima Immannezhad

**Affiliations:** 1 Psychiatry and Behavioral Sciences, Stanford University, Palo Alto, USA; 2 Psychiatry and Behavioral Sciences Research Center, Mashhad University of Medical Sciences, Mashhad, IRN; 3 Pediatrics, Mashhad University of Medical Sciences, Mashhad, IRN

**Keywords:** mania, encephalitis, psychiatric disease, covid-19 encephalitis, catatonia

## Abstract

There is growing evidence that coronavirus disease-2019 (COVID-19) infection may have various neuropsychiatric manifestations and long-term outcomes. In this article, the authors report a rare case of a 16-year-old male with no previous history of psychiatric illness who presented with an acute manic episode, including laughing for no evident reason, talking to himself, isolation, irritability, sleeplessness, decreased appetite, prolonged staring episodes, having delusions about being harmed or controlled, and aggression. Despite initiating outpatient treatment with a mood stabilizer and antipsychotic for presumed bipolar disorder with psychotic features, his symptoms worsened, and he became catatonic with a decreased level of consciousness, leading to his hospitalization on day 10. Although he had not shown typical evidence of infection with COVID-19 in the days leading up to or during his hospitalization and his initial COVID-19 test was negative, his COVID-19 test was positive on day 14, and his chest X-ray showed infiltrations. His acute manic symptoms and catatonia were identified to be associated with COVID-19 encephalitis after excluding other causes. He responded well to treatment with lorazepam for catatonia and a course of intravenous immunoglobulin, methylprednisolone, and remdesivir for COVID-19 encephalitis. This case demonstrates the workup and treatment of a rare neuropsychiatric manifestation of COVID-19 encephalitis in an adolescent, which started with no past psychiatric history and no typical symptoms of COVID-19 infection.

## Introduction

Encephalitis is an inflammatory disease of the brain caused by infections, autoimmune diseases, or medications. It often becomes characterized by its initial psychiatric manifestations [1]. In contrast to the adult population, the psychiatric manifestations of encephalitis in the pediatric population may present more frequently as manic symptoms rather than as psychosis [2].

Both coronavirus infections of severe acute respiratory syndrome coronavirus 2 (SARS-CoV-2) (COVID-19) and the previous SARS-CoV are associated with the elevation of pro-inflammatory cytokines and a cytokine storm of chemokines [3]. Many psychiatric disorders are characterized by inflammation, and their treatments have variable anti-inflammatory properties. Since inflammation is a common contributing factor in psychiatric disorders as well as SARS-CoV-2, there is a possibility that the treatment of either condition may affect the response to pharmacological treatment and disease progression [3].

In this article, the authors report a case of an adolescent with COVID-19 encephalitis presenting with manic-like symptoms and catatonia. The patient did not show typical symptoms of COVID-19 infection for about two weeks into his disease.

## Case presentation

A 16-year-old male presented with acute-onset symptoms including laughing for no evident reason, talking to himself, isolation, irritability, decreased need for sleep, decreased appetite, prolonged staring episodes, having delusions about being harmed or controlled, and an episode of conflict and aggression towards his family members.

The patient had no history of medical or psychiatric illness or substance use. He was raised by a single mother, and his father’s medical and psychiatric history was unknown. However, his maternal uncle was receiving medication for schizoaffective disorder, and his mother and older brother had type I diabetes.

The patient’s behavior changes had continued for over one week. He was referred to a psychiatrist and was treated as an outpatient for an acute manic episode of bipolar disorder with carbamazepine and chlorpromazine tablets due to the limited availability of other medications in his small town in Iran.

However, on day 10, the patient’s symptoms worsened, and he entered a catatonic state notable for complete mutism, negative acts, posturing, staring, rigidity, waxy flexibility, and decreased level of consciousness. He was admitted to a community hospital for treatment of catatonia. On admission, the patient was febrile with a temperature of 38.8°C. Other vital signs included blood pressure of 110/70 mmHg, respiratory rate of 12 breaths/minute, pulse rate of 72 beats/minute, and SpO2 of 92% on room air. His serum electrolytes, complete blood count, liver and kidney function tests, erythrocyte sedimentation rate (ESR), C-reactive protein (CRP), thyroid function tests, urinalysis and urine culture, lumbar puncture, chest computed tomography (CT), brain magnetic resonance imaging (MRI), and electroencephalogram (EEG) were reportedly normal, and his COVID-19 PCR test was initially negative.

Infectious disease specialists were consulted to rule out meningitis and other infectious diseases. An ophthalmology consult was performed to rule out the possibility of Kayser-Fleischer ring and Wilson's disease. A rheumatologist was consulted to rule out rheumatic and autoimmune diseases, and none of them reported any pathological findings.

After ruling out medical causes and not observing any improvement in the patient’s symptoms, the patient was initially diagnosed with bipolar disorder with a presentation of catatonia by a clinician in his small town and was transferred to a psychiatric hospital. He was orally treated with aripiprazole 15 mg daily and lorazepam 6 mg daily. Additionally, he received supportive nutritional treatments such as olive oil, serum therapy, and feeding through gavage since he had lost weight, MV Syrup (multivitamin multimineral and antioxidant syrup), and zinc syrup. As his symptoms worsened on day 12 and his level of consciousness decreased, he was transferred to the intensive care unit and underwent three electroconvulsive therapy (ECT) sessions.

On day 14 from the start of his symptoms, due to not showing a proper medical response, he was transferred from the small town to a tertiary psychiatric hospital in a larger city. On the way to the hospital, he had a suspected seizure and received an injection of midazolam, after which for about one hour, the patient's catatonia symptoms resolved dramatically.

Based on consultation with a pediatric neurologist, with clinical suspicion of COVID-19 encephalitis, he was transferred to the neurology department of an academic hospital. On this admission, his encephalitis panel for autoimmune diseases including anti-N-methyl-D-aspartate (NMDA)-receptor encephalitis and his EEG for anti-NMDA-receptor encephalitis were both normal. His abdominal ultrasound was negative for paraneoplastic encephalitis.

His cerebrospinal fluid (CSF) was negative for hepatitis C virus, Epstein-Barr virus, and herpes simplex encephalitis. His blood test was negative for human immunodeficiency virus. Aside from fever, the patient had no typical evidence of COVID-19 disease in the days leading up to hospitalization, and his family members were fully vaccinated against COVID-19. However, the patient himself was unvaccinated, and his sister, with whom he had recent contact, tested positive for COVID-19 at the time of the patient's onset of neuropsychiatric symptoms. On day 14, the patient’s COVID-19 test was positive, and his chest X-ray showed infiltrations. His EEG showed moderate dysrhythmia and his repeat EEG within one week showed an extreme delta brush pattern. His CSF was normal for protein and glucose, but its lymphocyte count was elevated to 15/mm^3^. The patient’s clinical presentation of acute manic-like symptoms and catatonia was determined to be associated with COVID-19 encephalitis after excluding other causes. His catatonia responded well to treatment with lorazepam, and he showed brief signs of improvement in his symptoms, including opening his eyes and briefly communicating and interacting with others, and he continued receiving 400 mg daily intravenous immunoglobulin (IVIG), pulse methylprednisolone 1 g daily, and remdesivir for five days.

Later, he was transferred to a psychiatric hospital for further treatment and underwent shock therapy, receiving six additional sessions of ECT. He was able to receive oral nutrition after four ECT sessions. Fecal and urinary incontinence, muscular rigidity, and his unusual body posture resolved, and he regained his full orientation to person, place, and time.

He gained about 2.5 kg in weight. He was able to establish meaningful eye contact and verbal communication with those around him and was again able to walk without assistance.

Given the patient's alteration in mental status, new-onset seizure, EEG changes, lymphocytic pleocytosis, fever, and positive COVID-19 test, the patient was diagnosed with COVID-19 encephalitis presenting with manic-like symptoms and catatonia. He was released from the hospital when his symptoms were well-controlled. He continued to receive maintenance treatment with oral olanzapine 10 mg daily and lorazepam 2 mg daily. Follow-up of the patient more than one year later showed a full recovery from the disease episode without taking any medication after the original treatment.

## Discussion

In some cases, a complete picture of a mood disorder may be seen in the course of a medical disease. Therefore, obtaining an accurate past and present medical history, physical examination, and, if necessary, paraclinical measures may be helpful. A comprehensive history and clinical workup may determine that the pathophysiological effects of a medical illness are resulting in an acute psychiatric presentation.

Encephalitis is an inflammatory disease of the brain due to several causes, the most common of which is viral encephalitis [4]. Encephalitis is an uncommon complication of COVID-19 [5,6]. Some studies have suggested that the SARS-CoV-2-induced immunologic response, rather than the infiltration of the virus itself, may create inflammatory damage in the CNS and cause the development of encephalitis [6-8]. Approximately 80% of patients diagnosed with encephalitis have early psychiatric manifestations, and approximately 40% may be initially admitted to a psychiatric unit [9]. Most encephalitis patients have no previous history of mental illness; thus, the first episode of a patient's psychiatric symptoms highlights the need for comprehensive physical examinations and medical workup [10]. Conventional psychotropic medications may help treat the psychiatric symptoms of primary medical illnesses, but their effects are transient and should be used with caution because 50% of patients who have encephalitis and are treated with these medications fail to receive adequate treatment results and may show pharmacological side effects [9,10].

For most viral encephalitis cases, treatment is only supportive, but intravenous immunoglobulins may be used as a preventative and therapeutic approach [11]. Studies show there are clinical benefits of using IVIG adjuvant therapy in children with viral encephalitis, including relieving some clinical symptoms such as spasms, reducing the duration of recovery time and return of consciousness, and resolving some neuropathic symptoms [12].

Cases of COVID-19 encephalitis and catatonia have been reported in adults [6,13-15]. However, reports of COVID-19 encephalitis in the pediatric population remain rare [16]. In our case of an adolescent with the presentation of manic-like symptoms and catatonia due to COVID-19 encephalitis, treatment of the underlying medical disease (encephalitis) along with the appropriate psychiatric treatment led to the patient's recovery.

## Conclusions

Medical or physical illnesses can present with various psychiatric symptoms, signs, and clinical features. Therefore, emergency medicine physicians, psychiatrists, neurologists, and internists should consider that acute psychiatric presentations may be caused by COVID-19 encephalitis. Thus, they should include COVID-19 encephalitis in the list of differential diagnoses for patients presenting with their first episode of psychiatric symptoms.
